# Reconsolidation of Maladaptive Memories as a Therapeutic Target: Pre-Clinical Data and Clinical Approaches

**DOI:** 10.3389/fpsyt.2014.00107

**Published:** 2014-08-19

**Authors:** Cristiano Chiamulera, Ina Hinnenthal, Alessia Auber, Mauro Cibin

**Affiliations:** ^1^Neuropsychopharmacology Laboratory, Sezione Farmacologia, Università di Verona, Policlinico “G.B. Rossi”, Verona, Italy; ^2^PhD School in Health Economics and Management, Catholic University of Sacred Heart, Rome, Italy; ^3^Health Services Research Laboratory, University of Siena, Siena, Italy; ^4^Healthcare System Unit 1 Imperiese, Imperia, Italy.; ^5^Addictive Behaviours Department, Local Health Authority, Dolo Venice, Italy

**Keywords:** substance use disorder, post-traumatic stress disorder, memory, reconsolidation, neuroscience

## Introduction

The retrieval of memories previously associated with a drug of abuse may increase the risk of relapse, even several years after abstinence (1). Similarly, memories associated to a traumatic event may cause an emotional disorder such as the post-traumatic stress disorder (PTSD). Recent neuroscience research has shown that memory retrieval induces a transient state during which memories are reactivated and then updated, modified, or even deleted (2). This transient process is called reconsolidation (3). Although the therapeutic potential of the manipulation of reconsolidation was raised only recently, the possibility to modify reactivated memories is somehow already applied in clinical interventions for substance use disorders (SUD) and PTSD.

Besides the forward translation from basic research to the clinic, there is now the opportunity to apply a “reverse engineering” approach from the complexity of the clinical setting to the underlying processes and mechanisms (4). This approach may make possible to better understand the efficacy of current clinical interventions by offering a conceptual framework for enabling potential implementation.

In this paper, we summarize the pre-clinical research evidence on the reconsolidation of emotional and appetitive memories. Then, we describe those human laboratory studies that demonstrated the possibility to interfere with memory reconsolidation. We also describe current clinical interventions for SUD and PTSD that act on memory processes and, finally, we report about on-going therapeutic experiences.

## Memory Retrieval and Reconsolidation

Until recently, it was believed that memory, after being stored, is stable forever. However, we now know that memories can be retrieved to strengthen, add, or remove information, and then updated through a process called reconsolidation (5, 6). Research both in humans and animals has revealed the specific mechanisms underlying reconsolidation. It has been shown that retrieval of specific information corresponds to the activation of molecular cascades at the level of neurons, in particular *N*-methyl-d-aspartate glutamate receptor (NMDARs) and β-adrenergic receptor leading to activation of signaling pathways and phosphorylation of transcription factors (7). When the mechanism is activated, it is vulnerable to being inhibited or altered by other concomitant information. The result is maintenance, disruption, or update of the retrieved information. The ability to retrieve and change some memories could be the basis for the intervention for disorders based on maladaptive memories, such as PTSD and SUD: new and safe information could be integrated into the initial memory trace so to modifying it.

In fact, associative learning and memory play a central role both in SUD and PTSD. The rewarding effects of drugs are associated with several stimuli: psychological states, people, objects, places, etc. As drug use progresses, it develops an association between these stimuli and drug effect. Even after years of abstinence, the retrieval of this associative memory may exert cue reactivity, that is psychological, physiological, and behavioral responses that may trigger relapse (8). One important diagnostic cluster of PTSD is re-experiencing the traumatic event as distressing memories and flashbacks triggered by reminders, including external cues (9).

## “Learning to Forget”: Post-Retrieval Extinction of Memory

Extinction is traditionally referred as the repeated exposure to conditioned stimuli in the absence of unconditioned stimuli. This unreinforced presentation of conditioned stimuli leads to the extinction of conditioned response. Extinction is not the forgetting of the original association, but it is new information about the association between the conditioned stimuli and the unconditioned stimulus. Some studies showed that an extinction therapy provided after the retrieval of a memory previously acquired in the laboratory alters the original memory (10). When memories are retrieved via a short exposure to stimuli associated to the traumatic event or to drug use, they enter into a vulnerable stage that lasts from 1 to 6 h after the retrieval. During this window of lability, the extinction (defined as post-retrieval-extinction) may be applied. Some pre-clinical studies both in animals (11, 12) and humans (12–15) have shown that the post-retrieval-extinction prevented relapse induced by stimuli previously associated with trauma or drugs [but for critical issues and limitations, see Ref. (16–20)]. These data suggested that extinction applied during the labile phase of memories interfere with the reconsolidation of original trauma or drug memories. It was seen that this type of manipulation interfered selectively with the memories that have been retrieved and did not alter other mnemonic traces. The effect of post-retrieval-extinction was specific on the process of reconsolidation. In fact, the therapy was ineffective when administered more than 6 h after the retrieval, when the memories are already reconsolidated and lability window is closed, or in the absence of retrieval manipulation [for a review, see Ref. (10)]. A study in a laboratory model of PTSD in human beings revealed that the effect of post-retrieval-extinction is long-lasting: there was no recovery on the original traumatic memory 1 year after the administration of the post-retrieval-extinction (14). A study on hospitalized heroin ex-addicts demonstrated the effectiveness of post-retrieval-extinction also on memories associated with drugs of abuse (12).

## Memory Manipulation in the Clinical Intervention for SUD and PTSD

Although several authors have recently suggested the manipulation of reconsolidation as a novel potential target for the treatment of PTSD or SUD, it should be noted that in clinical practice, there are already approaches that are based on memory retrieval of traumatic events or of drug-related events. It is therefore likely that the manipulation of the pathological memories via modulation of retrieval/reconsolidation processes is already a component of some clinical interventions. Recently, Gorman and Roose (21) speculated that “*the modification of old memories reactivated by adding new elements of understanding, resulting in obtaining a version less pathogenic is a crucial part of what happens in the psychoanalytic process*.” They recognized that “*important similarities between the ability to extinguish a memory of fear in the laboratory through blockade of reconsolidation and the specific situation of psychoanalytic psychotherapy, in which the material removed is reactivated through the transference, reworked consciously and therefore subject to repression and new relegated the unconscious*.”

Other therapeutic approaches could be re-interpreted as an application of the post-retrieval-extinction. The “Prolonged Exposure Therapy” is an approach to PTSD based on the reactivation of trauma memory that has been validated in several studies (22). This approach was tested in a comorbid PTSD/SUD patient population with a randomized trial in which a group received only the usual treatment for SUD, while the other group received a concomitant treatment of trauma exposure. The latter group showed an improvement in PTSD symptoms, but not differences in the severity of SUD. The Authors suggested the possibility of applying the therapy of trauma exposure in subjects with SUD without the risk of worsening the dependence (23).

The “desensitization eye movement and reprocessing” (EMDR) is a therapeutic practice for the treatment of PTSD firstly proposed by Shapiro (24). This approach combines the retrieval of traumatic memories with saccadic eye movements and other forms of bilateral stimuli, for example, auditory or tactile. The effectiveness of EMDR is explained as the result of inserting new information in the traumatic memory. Controlled studies have shown that saccadic eye movements reduced the emotional swings and increased attention and ability to reactivate the episodic memory. EMDR may work by changing dysfunctional stored information to a more adaptive way. This approach is an intervention for PTSD that is based on modification of retrieved memory and could be applied as a set of standardized procedures and protocols in a reproducible setting. Based on these results, Shapiro proposed a protocol (25) in which both traumatic memories and components specifically related to addictive behavior were retrieved. Hase et al. (26) reported the application of EMDR in hospitalized alcoholics with a study on randomized vs. control groups treated with traditional interventions. The craving for alcohol significantly decreased in the group treated with EMDR more than in the control group, both during the intervention and at 1 month later. Noteworthy, only the memory associated to alcohol, and not to traumatic memories, was actively recalled in the study. Authors concluded that remodeling of alcohol memory could occur independently from that of traumatic memories, even in patients with comorbid PTSD/alcoholism.

It has been recently proposed the potential usefulness of applying the therapeutic interventions for PTSD to addiction (27–29). This approach is based on two assumptions: (1) some of those with type I dependence appears to have a history of traumatic events, with a use of substance as an attempted PTSD self-treatment; (2) alcohol and drugs induce a traumatic-like situation, i.e., as a separation between emotional and cognitive functions.

We have applied this type of model in the residential program “Villa Soranzo,” Italy (30). This type of approach unlocks traumatic memories using EMDR, emotional approaches, body-work, art-therapy, and other non-verbal techniques, in small specific therapeutic trauma groups (8–12 patients) within the traditional interventions for addiction, such as relapse prevention and anti-craving pharmacotherapy (30). The cognitive processing is immediately afterward mainly duty of the individual therapeutic sessions. In this “Soranzo model,” unlike Mills et al. (23), the memory of the substance use experiences and of their emotional associations is considered an integral part of PTSD intervention. This program has been applied to both subjects with SUD only, particularly alcohol and cocaine, and in patients with comorbid PTSD/SUD.

The results, based on our clinical experience and follow-up at 6, 12, and 18 months, showed greater compliance to therapeutic program and to instructions for aftercare, a lower number of relapses and lapses, and a more complete elaboration by the patient of the issues underlying the addiction, particularly traumatic events (30). For patients with a comorbid PTSD, we also observed the reduction of specific symptoms.

## The Perspective of “Reverse Engineering”

An intervention based on retrieval/reconsolidation may have greater effectiveness, especially as far as inter-individual variability and risk of relapse are concerned, compared to existing approaches. It could be the first prototypic example of a treatment that addresses the pathogenic mechanisms of addiction allocated in the memory of the patient. A treatment of this type should include a retrieval phase in which each patient retrieve specific individual memories associated to both drug and traumatic experiences, context, or discrete cues (12, 31, 32). During the following phase of reconsolidation, within 1 to 6 h, memories could be associated with new non-drug-related information. This type of treatment should be given: (i) when the patient is able to feel his/her emotions and craving and (ii) in a condition that does not allow the patient to relapse (e.g., inpatient). The first point is much less obvious than it seems: most of the patients do not show craving and do not feel emotions if they are currently using the drug to which they are addicted. Those patients who experience craving even if they are not abstinent tend to seek for the drug, eventually resulting in drug-taking. In order to apply the retrieval/reconsolidation treatment, it is therefore necessary that patients must be abstinent for at least a week. Regarding the second point, we suggest to perform this type of treatment in a residential setting, as actually happened in the trials reported above.

The proposed model for comorbid PTSD/SUD in which the common element is the memory, could provide a new epistemological model useful for the understanding of other psychiatric conditions. It opens up a horizon by unthinkable developments: a new psychotherapy based on neuroscientific findings as a “common core” of psychopathology and diagnostic classification.

## Author Contributions

All Authors contributed to and have approved the final manuscript.

## Conflict of Interest Statement

Cristiano Chiamulera received grants and has consulted for Italian Cancer League (LILT), acted as a board member for Intermeeting SNC provider, and received teaching honoraria from Co.Ge.S. Società Cooperativa Sociale. Ina Hinnenthal, Alessia Auber, and Mauro Cibin received teaching honoraria from Co.Ge.S Società Cooperativa Sociale.

## References

[1] O’Brien CP Childress AR McLellan AT Ehrman R A learning model of addiction. Res Publ Assoc Res Nerv Ment Dis (1992) 70:157–77 1535929

[2] Milton AL Everitt BJ The persistence of maladaptive memory: addiction, drug memories and anti-relapse treatments. Neurosci Biobehav Rev (2012) 36:1119–39 10.1016/j.neubiorev.2012.01.002 22285426

[3] Lewis DJ Psychology of active and inactive memory. Psychol Bull (1979) 86:1054–83 10.1037/0033-2909.86.5.1054 386401

[4] Sinha R Shaham Y Heilig M Translational and reverse translational research on the role of stress in drug craving and relapse. Psychopharmacology (2011) 218(1):69–82 10.1007/s00213-011-2263-y 21494792 PMC3192289

[5] Nader K Shafe GE Ledoux JE The labile nature of consolidation theory. Nat Rev Neurosci (2000) 1:216–9 10.1038/35044580 11257912

[6] Sara SJ Retrieval and reconsolidation: toward a neurobiology of remembering. Learn Mem (2000) 7:73–84 10.1101/lm.7.2.73 10753974

[7] Tronson NC Taylor JR Addiction: a drug-induced disorder of memory reconsolidation. Curr Opin Neurobiol (2013) 23:573–80 10.1016/j.conb.2013.01.022 23415831 PMC3677957

[8] Chiamulera C Cue reactivity in nicotine and tobacco dependence: a “multiple-action” model of nicotine as a primary reinforcement and as an enhancer of the effects of smoking-associated stimuli. Brain Res Brain Res Rev (2005) 48:74–97 10.1016/j.brainresrev.2004.08.005 15708629

[9] American Psychiatric Association. Diagnostic and Statistical Manual of Mental Disorders. 5th ed Arlington, VA: American Psychiatric Publishing (2013).

[10] Auber A Tedesco V Jones CE Monfils MH Chiamulera C Post-retrieval extinction as reconsolidation interference: methodological issues or boundary conditions? Psychopharmacology (2013) 226:631–47 10.1007/s00213-013-3004-1 23404065 PMC3682675

[11] Flavell CR Barber DJ Lee JL Behavioural memory reconsolidation of food and fear memories. Nat Commun (2011) 2:504 10.1038/ncomms1515 22009036 PMC3516828

[12] Xue YX Luo YX Wu P Shi HS Xue LF Chen C A memory retrieval-extinction procedure to prevent drug craving and relapse. Science (2012) 336:241–5 10.1126/science.1215070 22499948 PMC3695463

[13] Monfils MH Cowansage KK Klann E LeDoux JE Extinction-reconsolidation boundaries: key to persistent attenuation of fear memories. Science (2009) 324:951–5 10.1126/science.1167975 19342552 PMC3625935

[14] Schiller D Monfils MH Raio CM Johnson DC Ledoux JE Phelps EA Preventing the return of fear in humans using reconsolidation update mechanisms. Nature (2010) 463:49–53 10.1038/nature08637 20010606 PMC3640262

[15] Oyarzún JP Lopez-Barroso D Fuentemilla L Cucurell D Pedraza C Rodriguez-Fornells A Updating fearful memories with extinction training during reconsolidation: a human study using auditory aversive stimuli. PLoS One (2012) 7:e38849 10.1371/journal.pone.0038849 22768048 PMC3387215

[16] Auber A Muthu Karuppasamy NS Pedercini M Bertoglio D Tedesco V Chiamulera C The effect of postretrieval extinction of nicotine Pavlovian memories in rats trained to self-administer nicotine. Nicotine Tob Res (2014). 10.1093/ntr/ntu110 25038771

[17] Chan WY Leung HT Westbrook RF McNally GP Effects of recent exposure to a conditioned stimulus on extinction of Pavlovian fear conditioning. Learn Mem (2010) 17:512–21 10.1101/lm.1912510 20884753 PMC2948891

[18] Costanzi M Cannas S Saraulli D Rossi-Arnaud C Cestari V Extinction after retrieval: effects on the associative and nonassociative components of remote contextual fear memory. Learn Mem (2011) 18:508–18 10.1101/lm.2175811 21764847

[19] Ma X Zhang JJ Yu LC Post-retrieval extinction training enhances or hinders the extinction of morphine-induced conditioned place preference in rats dependent on the retrieval-extinction interval. Psychopharmacology (2012) 221:19–26 10.1007/s00213-011-2545-4 22048130

[20] Kindt M Soeter M Reconsolidation in a human fear conditioning study: a test of extinction as updating mechanism. Biol Psychol (2013) 92(1):43–50 10.1016/j.biopsycho.2011.09.016 21986472

[21] Gorman JM Roose SP The neurobiology of fear memory reconsolidation and psychoanalytic theory. J Am Psychoanal Assoc (2011) 59:1201–20 10.1177/0003065111427724 22080504

[22] Committee on Treatment of Posttraumatic Stress Disorder. Treatment of Posttraumatic Stress Disorder: An Assessment of the Evidence. Washington, DC: National Academies Press (2008).

[23] Mills KL Teesson M Back SE Brady KT Baker AL Hopwood S Integrated exposure-based therapy for co-occurring posttraumatic stress disorder and substance dependence: a randomized controlled trial. JAMA (2012) 308:690–9 10.1001/jama.2012.9071 22893166

[24] Shapiro F Eye movement desensitisation: a new treatment for posttraumatic stress disorder. J Behav Ther Exp Psychiatry (1989) 20:211–7 10.1016/0005-7916(89)90025-6 2576656

[25] Shapiro F Vogelmann-Sine S Sine LF Eye movement desensitization and reprocessing: treating trauma and substance abuse. J Psychoactive Drugs (1994) 26:379–91 10.1080/02791072.1994.10472458 7884600

[26] Hase M Shallmayer S Sack M EMDR reprocessing of the addiction memory: pretreatment, posttreatment and 1-month follow-up. J EMDR Pract Res (2008) 2:170–9 10.1891/1933-3196.2.3.170

[27] Hinnenthal I Laki Z Ardissone G Psicotraumatologia e neuroplasticità. Presupposti teorici per la gestione clinica nel trattamento residenziale di alcolisti con poliabuso. In: Lucchini A Nava F Manzato E, editors. Buone Pratiche e Procedure Terapeutiche Nella Gestione del Paziente Alcolista. Milano: Franco Angeli (2008). p. 103–9

[28] Hinnenthal I Asam EC Psicotraumatologia. In: Hinnenthal I Cibin M, editors. Il Trattamento Residenziale Breve Delle Dipendenze da Alcol e Cocaine: Il Modello Soranzo. Torino: SEEd (2011). p. 123–34

[29] Hinnenthal I Villa Soranzo non è un luogo, è un’idea: aspetti pratici, clinici e di supervision. In: Hinnenthal I Cibin M, editors. Il Trattamento Residenziale Breve Delle Dipendenze da Alcol e Cocaine: Il Modello Soranzo. Torino: SEEd (2011). p. 187–98

[30] Cibin M Jester A Leonardini L Lugato E Papanastasatos G Transnational catalogue of intervention options for young polidrug users. Strategic European Inventory on Drugs. E.U. Executive Agency for Health and Consumers. (2010). Available from: http://lnx.ceisdonmilani.com/seidproject/Transnational_Catalogue.pdf

[31] Taylor JR Olausson P Quinn JJ Torregrossa MM Targeting extinction and reconsolidation mechanisms to combat the impact of drug cues on addiction. Neuropharmacology (2009) 56(Suppl 1):186–95 10.1016/j.neuropharm.2008.07.027 18708077 PMC2635342

[32] Hinnenthal I Spolaor G Cibin M Nante N Schmidt R Simboli, metafore e immagini nel trattamento psicoterapeutico del trauma e dell’addiction. Medicina Dipendenze (2013) 12:31–6

